# Error in Byline

**DOI:** 10.1001/jamahealthforum.2026.2518

**Published:** 2026-07-10

In the Original Investigation titled “Tort Immunity and Nursing Home Staffing,”^1^ published online on June 1, 2026, there was an error in the byline. The third author’s name should appear as David Powell, PhD. This article has been corrected online.
